# Individualized supplement of water-soluble vitamins: the influence of inflammation and renal function on circulating concentrations in critically digestive disease patients

**DOI:** 10.3389/fimmu.2025.1583568

**Published:** 2025-07-25

**Authors:** Jingjing Wang, Jing Yan, Qi Chen, Linlin Shi, Ying Wang, Xiaoxiao Tian, Yumei Qi, Guoxun Li, Hailong Cao

**Affiliations:** ^1^ Department of Nutrition, the Third Central Hospital of Tianjin, National Medical Quality Control Center of Clinical Nutrition, Tianjin Key Laboratory of Extracorporeal Life Support for Critical Diseases, Artificial Cell Engineering Technology Research Center, Tianjin Institute of Hepatobiliary Disease, Tianjin, China; ^2^ Department of Gastroenterology and Hepatology, Tianjin Medical University General Hospital, National Key Clinical Specialty, Tianjin Institute of Digestive Diseases, Tianjin Key Laboratory of Digestive Diseases, Tianjin, China; ^3^ Department of Nutrition, the Second Affiliated Hospital, Air Force Medical University, Xi’an, China

**Keywords:** water-soluble vitamins, deficiency, accumulation, inflammation, renal function, critical digestive disease

## Abstract

**Background:**

Existing studies have shown the association of circulating vitamin and disease outcome. The study aimed to elucidate individual response of plasma water-soluble vitamins after supplement by PN in critically digestive disorder patients.

**Methods:**

We measured the plasma levels of nine water-soluble vitamins (i.e., C, B1, B2, B3, B5, B6, B7, B9, and B12) in consecutive 478 hospitalized critically digestive disease patients receiving identical vitamin-supplemented by PN. Univariate and multifactorial logistic regression analysis were used to evaluated vitamins deficiency and accumulation. The receiver operating characteristic (ROC) curves were used to predict vitamin abnormalities. Furthermore, restricted cubic spline (RCS) was used to analyze the National Health and Nutrition Examination Survey (NHANES) database (2003-2020). Additionally, plasma vitamins levels were contrastive analyzed after PN.

**Results:**

There were high prevalence of vitamin C and B9 deficiency (79.71% and 78.45%) but vitamin B2, B5, and B6 accumulation (34.52%, 12.13%, and 11.09%). Multivariate logistic regression analysis revealed that inflammation is an independent risk factor for vitamin C and B9 deficiency, whereas renal dysfunction is an independent risk factor for vitamin B2, B5, and B6 accumulation. The areas under the ROC curves predicting vitamin C, B9 deficiency and vitamin B2, B5, B6 accumulation were 0.80, 0.75, 0.69, 0.79, and 0.89, respectively. The NHANES database further confirms our conclusion. Conventional vitamin supplementation may not efficiently alleviate vitamin C and B9 deficiency in patients with high inflammation, however, it may accelerate plasma vitamin B2, B5, and B6 accumulation with renal dysfunction.

**Conclusion:**

Water-soluble vitamin levels were associated with inflammation and renal function. For high inflammation, vitamin C and B9 doses may need to exceed standard levels. In renal impairment, avoid indiscriminate B2, B5, and B6 use; if needed, use alternate-day dosing or lower doses.

## 1 Introduction

Vitamins are essential micronutrients that cannot be synthesized and must be obtained from food (1). According to their solubility, vitamins can be divided into two types: water-soluble and lipid-soluble. Nine water-soluble vitamins have been identified thus far: vitamin C (ascorbic acid) and the B vitamin family members, namely B1 (thiamine), B2 (riboflavin), B3 (niacin), B5 (pantothenic acid), B6 (pyridoxine), B7 (biotin), B9 (folate), B12 (cobalamin) (2). Because water-soluble vitamins are not stored within the body, their deficiency is highly prevalent, which can affect disease outcomes. In cases of over-supplementation, water-soluble vitamins are excreted via urine. However, in patients receiving parenteral nutrition (PN), vitamins are not digested and absorbed through the gastrointestinal tract; rather, they enter the bloodstream directly. Whether water-soluble vitamin accumulation occurs in patients with PN remains unclear. B vitamins are typically supplemented in a complex preparation intravenously or via enteral nutrition. Vitamin abnormalities are highly prevalent among hospitalized patients (3–5); however, most research thus far has focused on vitamin deficiencies rather than vitamin accumulation.

With the improvement of living standards, starvation-related malnutrition prevalence has decreased. Nevertheless, disease-related malnutrition (DRM) is gradually gaining attention (6). According to the European Society of Clinical Nutrition and Metabolism (ESPEN), malnutrition can be defined as “a state resulting from lack of intake or uptake of nutrition that leads to altered body composition (decreased fat-free mass) and body cell mass leading to diminished physical and mental function and impaired clinical outcome from disease” (7). The ESPEN classifies malnutrition into DRM with or without infilammation and malnutrition or undernutrition without disease. Based on the presence of inflammation, DRM can be categorized into inflammation and noninflammation DRM. Accumulating evidence indicates that inflammation is a key driver of DRM, resulting in loss of appetite, reduced food consumption (8–10), accelerated muscle loss (11), and insulin resistance (12). Malnutrition or undernutrition, overweight, obesity, micronutrient abnormalities, and refeeding syndrome are generally considered nutritional disorders. Moreover, specific micronutrient deficiencies are often associated with malnutrition or undernutrition. Hence, we hypothesized that inflammation affects the disorder of micronutrients, particularly water-soluble vitamins, which leads to malnutrition.

Water-soluble vitamin abnormalities may result from changes in food intake, nutrient absorption, nutrient loss, nutrient demand, or medication use, individually or in combination (13). Patients with severe digestive disorders generally exhibit restricted dietary intake and impaired nutrient digestion/absorption, significantly increasing the risk of deficiencies in water-soluble vitamins and other micronutrients, which warrants significant clinical attention. Water-soluble vitamin requirements vary with age, sex, and disease status. A vitamin deficiency affects metabolism, resulting in malnutrition, immunocompromise, disease predisposition, and poor clinical prognosis (14–16). However, water-soluble vitamin metabolism in patients with renal disorders remains underexplored, and the findings related to plasma water-soluble vitamin levels in kidney dialysis patients have been inconsistent. As such, the water-soluble vitamin accumulation of such patients remains unclear. The benefits and safety of water-soluble vitamin supplementation in patients receiving PN remain unclear. Triona et al. reported that 100%, 80%, and 39% of the children on dialysis had high vitamin B6, vitamin B12, and folate levels, respectively (17). However, Oguzhan et al. found that of patients with end-stage renal disease, 24.7% and 35.1% had vitamin B1 and B6 deficiencies, respectively (18).

In the present study, we assessed the plasma levels of water-soluble vitamins in patients with critical digestive diseases receiving PN and explored the factors affecting these levels to provide evidence for precise PN implementation in the future.

## 2 Materials and methods

### 2.1 Study population and data collection

**Figure 1:**
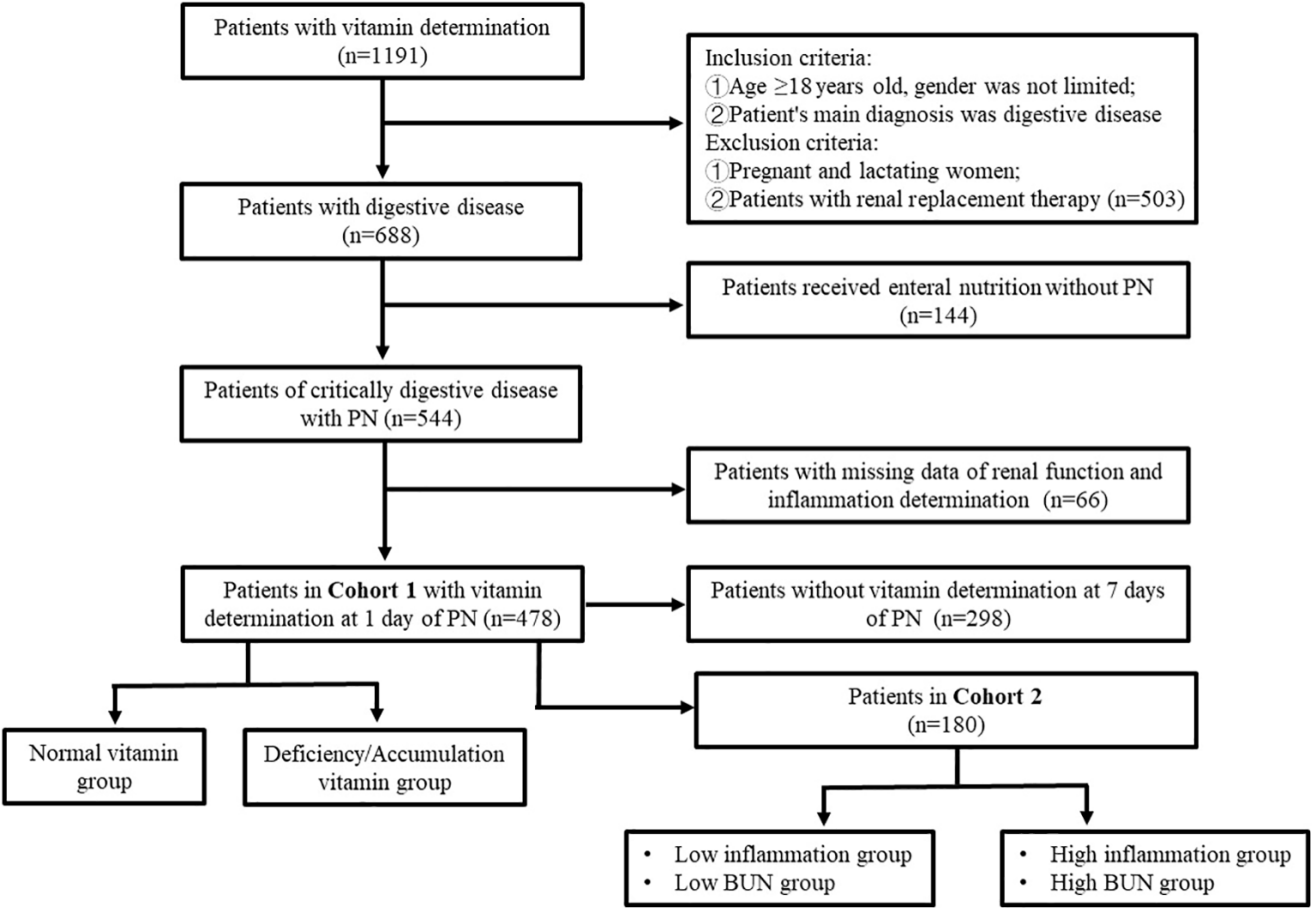
Flow diagram for the selection of patients in the study.

In total, 1,191 inpatients suspected of having vitamin deficiency or receiving PN underwent plasma water-soluble vitamin assessment in Tianjin Third Central Hospital from September 2021 to July 2023. Of them, 478 patients with severe digestive disorders with complete clinical data were finally enrolled in the study. The plasma water-soluble vitamin levels of 180 patients on PN days 1 and 7 were compared. The flow of patient selection and exclusion is illustrated in **Figure 1**. This study was approved by the Ethics Committee of Tianjin Third Central Hospital (No. IRB2024-01-0003).

#### 2.1.1 Nutrition assessment and treatment

At the study hospital, all inpatients undergo nutritional risk screening based on Nutrition Risk Screening (NRS) 2002 at hospitalization. If the NRS 2002 score is ≥3, a nutritionist conducts nutritional assessments, including physical measurements and dietary surveys, in the patients. Malnutrition and its severity are diagnosed according to the Global Leadership Initiative on Malnutrition (GLIM) criteria (19). Nutritional treatment is administered via the appropriate route based on the disease type (20). Enteral nutrition is recommended to patients without contraindications, whereas PN is administered to patients who cannot tolerate enteral nutrition (21). All-in-one admixtures administered to the patients contain compound amino acid injection (18AA), lipid emulsion (MCT/LCT), glucose (5%, 10%, 20%), electrolyte solution (sodium chloride injection, potassium chloride injection, calcium carbonate injection, magnesium sulfate injection, sodium glycerophosphate injection), trace elements (Multi-trace Elements Injection II; FRESENIU KABI SSPC, China), water-soluble vitamins (Verapamil Hydrochloride Tablets; FRESENIU KABI SSPC), and lipid-soluble vitamins (Fat-soluble Vitamin Injection II; FRESENIU KABI SSPC). The nutritionist adjusts the proportion of each nutrient according to the needs of the patients. The amount of water-soluble vitamins (i.e., Verapamil Hydrochloride Tablets) supplied is determined by total energy (22): 5–8 mL when total energy < 750 kcal, whereas 10 mL when total energy ≥ 750 kcal.

Patients screened in this study were fasting or had insufficient oral intake to meet daily nutritional requirements, thus requiring PN. Parenteral nutrition doses were determined by actual food intake, with this study's participants receiving PN > 750 kcal with 10 mL verapamil hydrochloride tablets (23) and 5 mL vitamin C injection (500 mg). Daily water-soluble vitamin supplements in PN were vitamin C (613 mg), vitamin B1 (3.1 mg), vitamin B2 (4.9 mg), vitamin B3 (40 mg), vitamin B5 (16.5 mg), vitamin B6 (4.9 mg), vitamin B7 (60 μg), vitamin B9 (0.4 mg), and vitamin B12 (5 μg).

#### 2.1.2 Blood biochemistry and water-soluble vitamin assessment

Blood samples were collected from the patients early in the morning on an empty stomach after nutritional therapy. Urine volume and water-soluble vitamin supplementation were recorded 1 day before blood sampling. All samples were processed for biochemical analysis at the biochemistry laboratory of the study hospital. White blood cell count (WBC), neutrophil count (NE), lymphocyte count (LYM), and hemoglobin (Hb) in the blood samples were quantified on the ADVIA-2120 autoanalyzer. Systemic Immunity-Inflammation Index (SII) was calculated as (platelet count × neutrophil count)/lymphocyte count. Plasma C-reactive protein (CRP), blood urea nitrogen (BUN), and blood creatinine (Cr) levels were measured on a light scattering turbidimeter (IMMAGE 800; Beckman). Blood albumin (ALB) and prealbumin (PA) levels were measured on the automatic biochemical analyzer MODULAR P800 (Roche). Blood uric acid (UA) levels were measured on the automatic biochemical analyzer TBA-120 by using specific kits from Shanghai Rongsheng Biology (China).

On PN day 1, the patients were offered a nutritionist consultation. To measure a baseline level of plasma vitamins, the blood was drawn on the morning of PN day 2. Plasma water-soluble vitamins were measured through ultrahigh performance liquid chromatography–tandem mass spectrometry (ACQUITY; Waters, USA) by using a kit from Yingsheng Biology (China).

### 2.2 Statistical analysis

SPSS (version 22.0), GraphPad Prism (version 8.2.1) and R version 4.4.1 were used for data analyses and visualization. Variable data distribution was evaluated using the Shapiro–Wilk test. Normally and nonnormally distributed data are expressed as means ± standard deviations and medians with interquartile ranges (IQRs), respectively; categorical variables are expressed as counts and percentages. Correlation analysis was conducted using a Spearman correlation coefficient and simple linear regression. Univariate and multivariate logistic regression analysis was performed to predict water-soluble vitamin deficiency and accumulation. To evaluate the power of prediction and the diagnostic performance for vitamin C and B9 deficiency and vitamin B2, B5, and B6 accumulation, we performed receiver operating characteristic (ROC) curve analysis and calculated the area under the ROC curve (AUC). To assess the shape of the relationship between vitamin B6 and BUN, we created restricted cubic spline plots. *P* < 0.05 was considered to indicate statistical significance.

## 3 Results

### 3.1 Patient characteristics and plasma water-soluble vitamin levels

**Table 1** presents the baseline characteristics of our patients. In total, 478 patients were included in this study [median age = 67.0 years; 323 (67.57%) male]. Of all patients, 61.72% were aged ≥65 years. All patients received PN at the same dosage of water-soluble vitamins described above. Patients were mainly diagnosed as having a digestive disorder, including an esophageal disease (n = 16; 3.35%), gastric disease (n = 56; 11.72%), intestinal disease (n = 149; 31.17%), hepatobiliary disease (n = 191; 39.96%), and pancreatic disease (n = 66; 13.81%).

**Table 1:** Patient characteristics.

Characteristic (n = 478)	Value
Age, years, median (IQR)	67.000 (61.000, 74.000)
<45 years, n (%)	24 (5.021)
45–65 years, n (%)	159 (33.264)
>65 years, n (%)	295 (61.715)
Sex, n (%)	
Female, n (%)	155 (32.427)
Male, n (%)	323 (67.573)
Disease type, n(%)	
Esophageal disease	16 (3.347)
Gastric disease	56 (11.715)
Intestinal disease	149 (31.172)
Hepatobiliary disease	191 (39.958)
Pancreatic disease	66 (13.807)

IQR, interquartile range.

**Figure 2:**
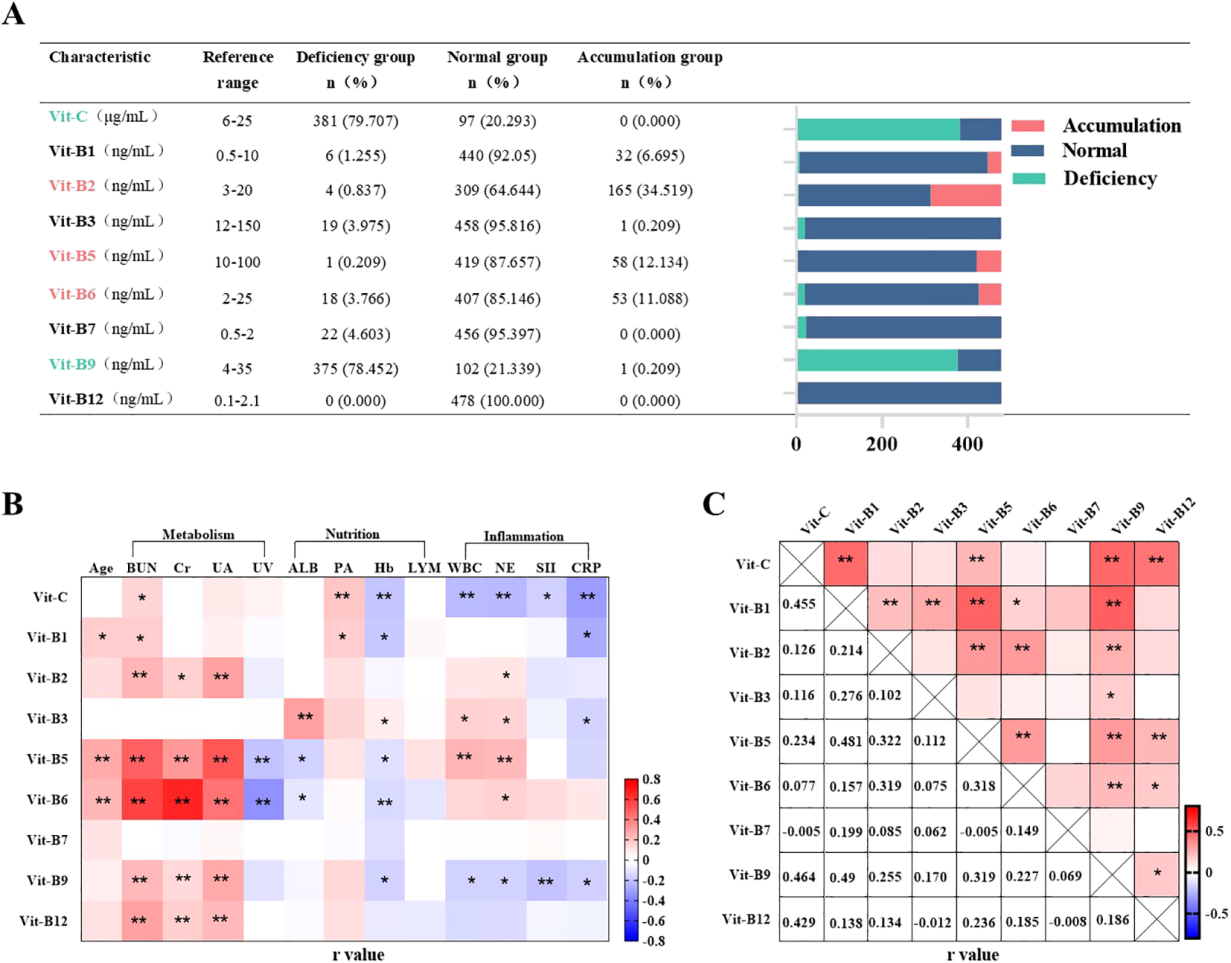
Incidence of abnormal plasma vitamin levels and correlation analysis. n=478. **(A)** Plasma water soluble vitamin levels of patients with PN for 1 day. Values of plasma water-soluble vitamins below the lower limit of the reference value were named “L-group”, above the upper limit of the reference value were named “H-group” and within the reference value range were named “N-group”. **(B, C)** Correlation analysis of plasma water-soluble vitamins in patients with PN. Pearson r = (–1, 1). *P <0.05, **P <0.01. BUN, blood urea nitrogen; Cr, blood creatinine; UA, blood uric acid; WBC, blood white blood cell count; NE, blood neutrophil count; LYM, blood neutrophil count; SII, systemic immune-inflammatory index; CRP, C-reactive protein; Hb, hemoglobin; ALB, blood albumin; PA, blood pre-albumin. UV, Urinary volume.

Patients with plasma water-soluble vitamin levels below the lower limit of, above the higher limit of, and within the reference range were categorized into L, H, and N groups, respectively. The L and H group patients were considered to indicate vitamin “deficiency” and “accumulation”, respectively. In this study, observed “deficiency” reflects biochemical deviations and may not align with clinical manifestations of vitamin deficiency (24). Most of our patients tended to be deficient in vitamin C (79.71%) and B9 (78.45%) but demonstrate vitamin B2 (34.52%), B5 (12.13%), and B6 (11.09%) accumulation (**Figure 2A**).

### 3.2 Correlation analysis of plasma water-soluble vitamin levels

We analyzed the relationships of plasma vitamin levels with the biomarkers of inflammation (WBC, NE, SII, and CRP), renal function (BUN, Cr, UA, and urine volume), and nutritional status (Hb, ALB, PA, and LYM) in all 478 patients. Pearson correlation analysis based on heatmaps showed that inflammation demonstrated a two-sided relationship: it was correlated negatively with plasma vitamin C, B9, and B1 levels but positively with plasma vitamin B2, B3, B5, and B6 levels. Plasma vitamin B7 and B12 levels were not correlated with inflammation. Renal-function biomarkers were positively correlated with the plasma levels of all water-soluble vitamins except vitamin B3 and B7. Among them, the daily urine volume was negatively correlated with vitamins B5 and B6. However, our selected nutritional status biomarkers did not appear to be correlated with plasma water-soluble vitamin levels. Notably, Hb was negatively correlated with plasma vitamin C, B1, B5, B6, and B9 levels (**Figure 2B**). Heatmaps demonstrated positive pairwise correlations among plasma water-soluble vitamin levels, with partial correlation coefficients ranging from 0.005 to 0.49. However, plasma vitamin B7 levels were not correlated to all other water-soluble vitamins (**Figure 2C**).

### 3.3 Analysis of abnormal plasma water-soluble vitamin levels

The incidence of abnormal vitamin C, B9, B2, B5, and B6 levels was relatively high in this study. Thus, we analyzed vitamin C and B9 deficiency and vitamin B2, B5, and B6 accumulation among our patients. According to the univariate logistic regression analysis, the factors significantly influencing vitamin C deficiency were age, BUN, WBC, NE, SII, CRP, and Hb (**Supplementary Table S1**); those significantly influencing vitamin B9 deficiency were age, sex, BUN, UA, WBC, SII, CRP, LYM, and Hb (**Supplementary Table S2**); those significantly influencing vitamin B2 accumulation were BUN, Cr, UA, urine volume, ALB and Hb (**Supplementary Table S3**); those significantly influencing vitamin B5 accumulation were age, BUN, Cr, UA, urine volume, WBC, and ALB (**Supplementary Table S4**); and finally, those significantly influencing vitamin B6 accumulation were age, BUN, Cr, UA, urine volume, WBC and ALB (**Supplementary Table S5**).

**Figure 3:**
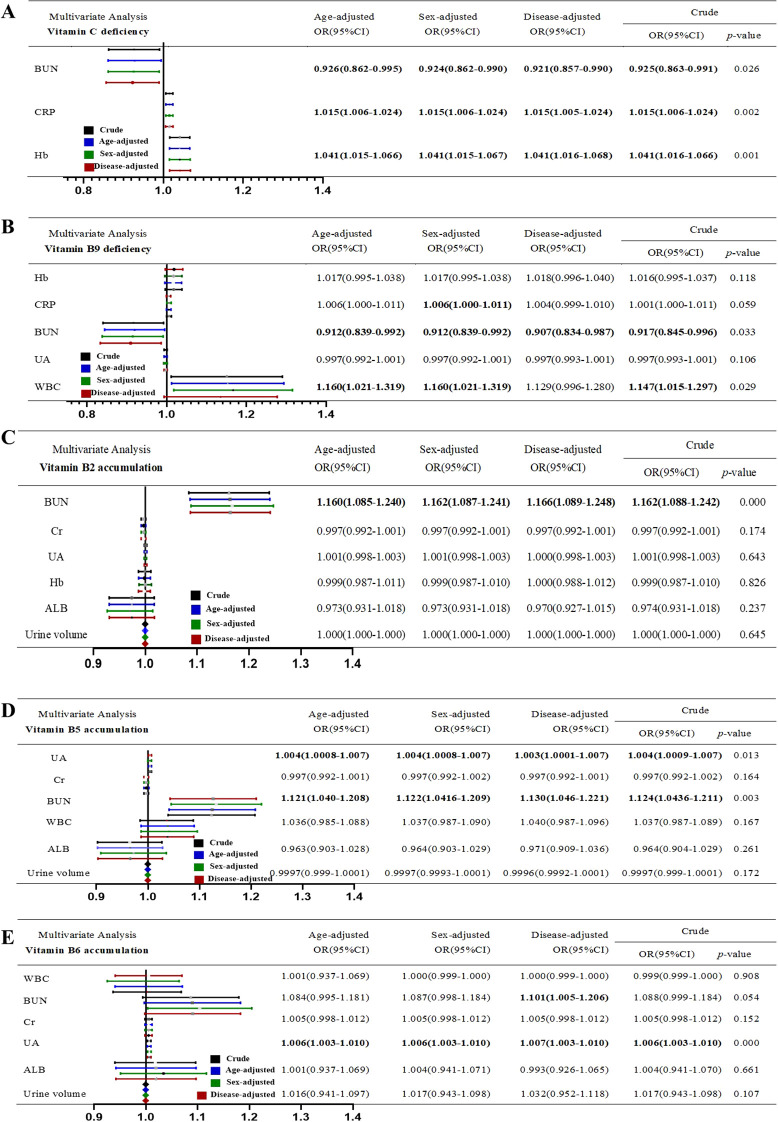
Multivariable logistic regression of risk or protective factors of Vitamin disorder. **(A)** Vitamin C deficiency. **(B)** Vitamin B9 deficiency. **(C)** Vitamin B2 accumulation. **(D)** Vitamin B5 accumulation. **(E)** Vitamin B6 accumulation. “Crude” means non-adjusted. The OR values were adjusted for age, sex and disease.

Next, we performed binary logistic regression analysis adjusted for age, sex, and disease type. The results demonstrated that for vitamin C and B9 deficiency, inflammation was an independent risk factor, whereas high BUN level was an independent protective factor. Moreover, high Hb level was noted to be an independent risk factor for vitamin C deficiency (**Figures 3A, B**). Multivariate logistic regression analysis revealed that renal dysfunction (high BUN or UA) was an independent risk factor for vitamin B2, B5, and B6 accumulation (**Figures 3C–E**).

### 3.4 Models to predict plasma water-soluble vitamin abnormalities

**Figure 4:**
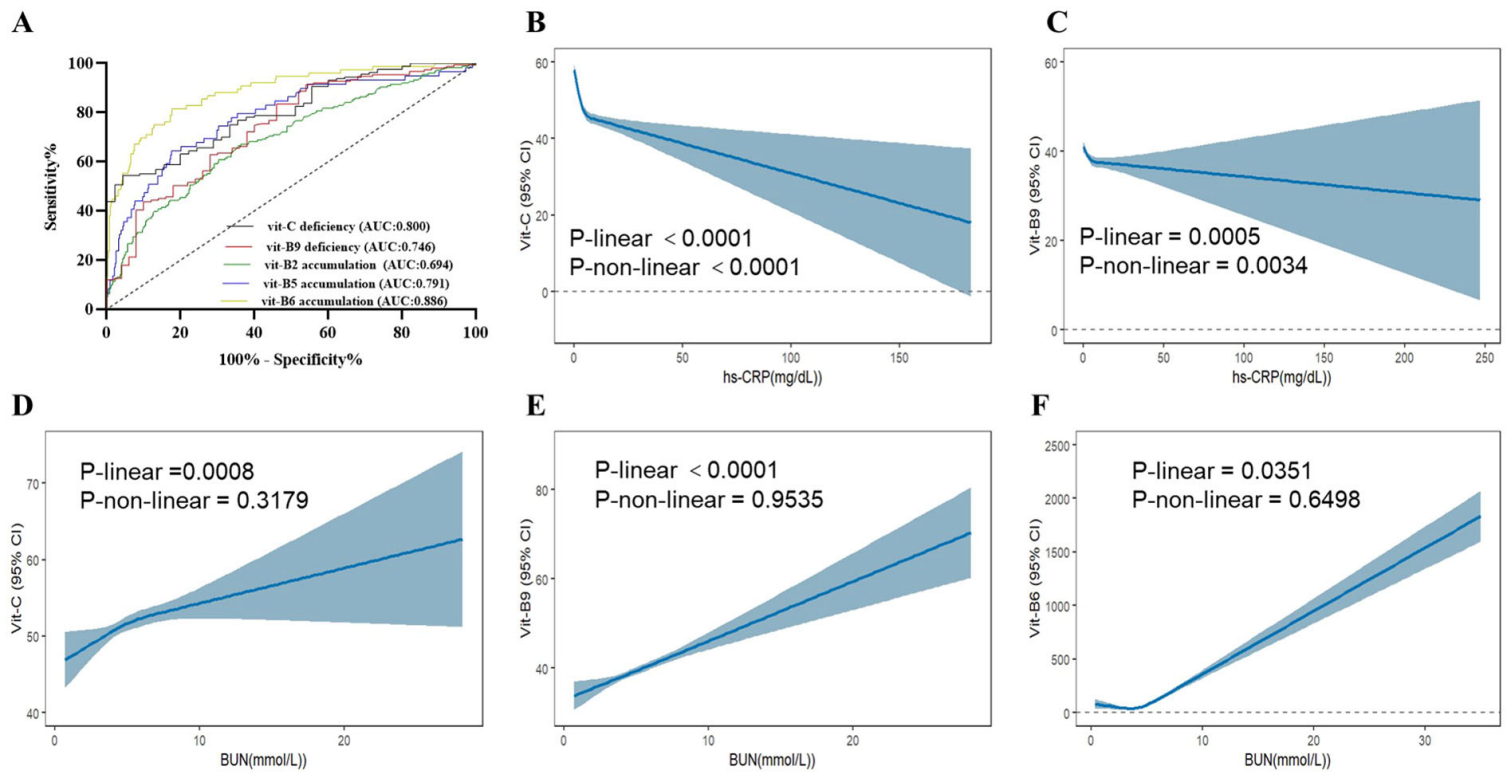
ROC of plasma water vitamin deficiency and accumulation abnormal and verified in the NHANES from 2003-2020**(A)** ROC curve in PN patients. AUC, area under the curve. **(B-F)** Analysis of serum vitamin C, B9 and B6 in the NHANES from 2003-2020. **(B)** Association between hs-CRP and vitamin C **(C)** Association between hs-CRP and vitamin B9. **(D)** Association between BUN and vitamin C **(E)** Association between BUN and vitamin B9. **(F)** Association between BUN and vitamin B6. hs-CRP, hypersensitive C-reactive protein.

Routine clinical protocols do not involve plasma water-soluble vitamin monitoring. However, inflammation and renal-function markers may predict disorders related to vitamin deficiency or accumulation. Here, we used various risk factors for vitamin abnormalities to build models predicting vitamin deficiency and accumulation. In particular, we combined BUN, CRP, and Hb to predict vitamin C deficiency (AUC = 0.80); Hb, CRP, BUN, UA, and WBC to predict vitamin B9 deficiency (AUC = 0.75); and BUN, Cr, UA, Hb, ALB, and urine volume to predict vitamin B2, B5, and B6 accumulation (AUCs = 0.69, 0.79, and 0.89, respectively; **Figure 4A**).

### 3.5 Validation based on National Health and Nutrition Examination Survey data

We next assessed the database of National Health and Nutrition Examination Survey (NHANES) among 2003–2020 participants. After matching and analyzing the NHANES database data, serum levels of vitamin C and B9 were found to be negatively correlated with hs-CRP, which strongly supports our conclusion. Meanwhile, vitamin C, B9, and B6 were found to be positively correlated with BUN, which aligns with our original conclusions (**Figures 4B-D**).

### 3.6 Plasma water-soluble vitamin changes after PN

**Figure 5:**
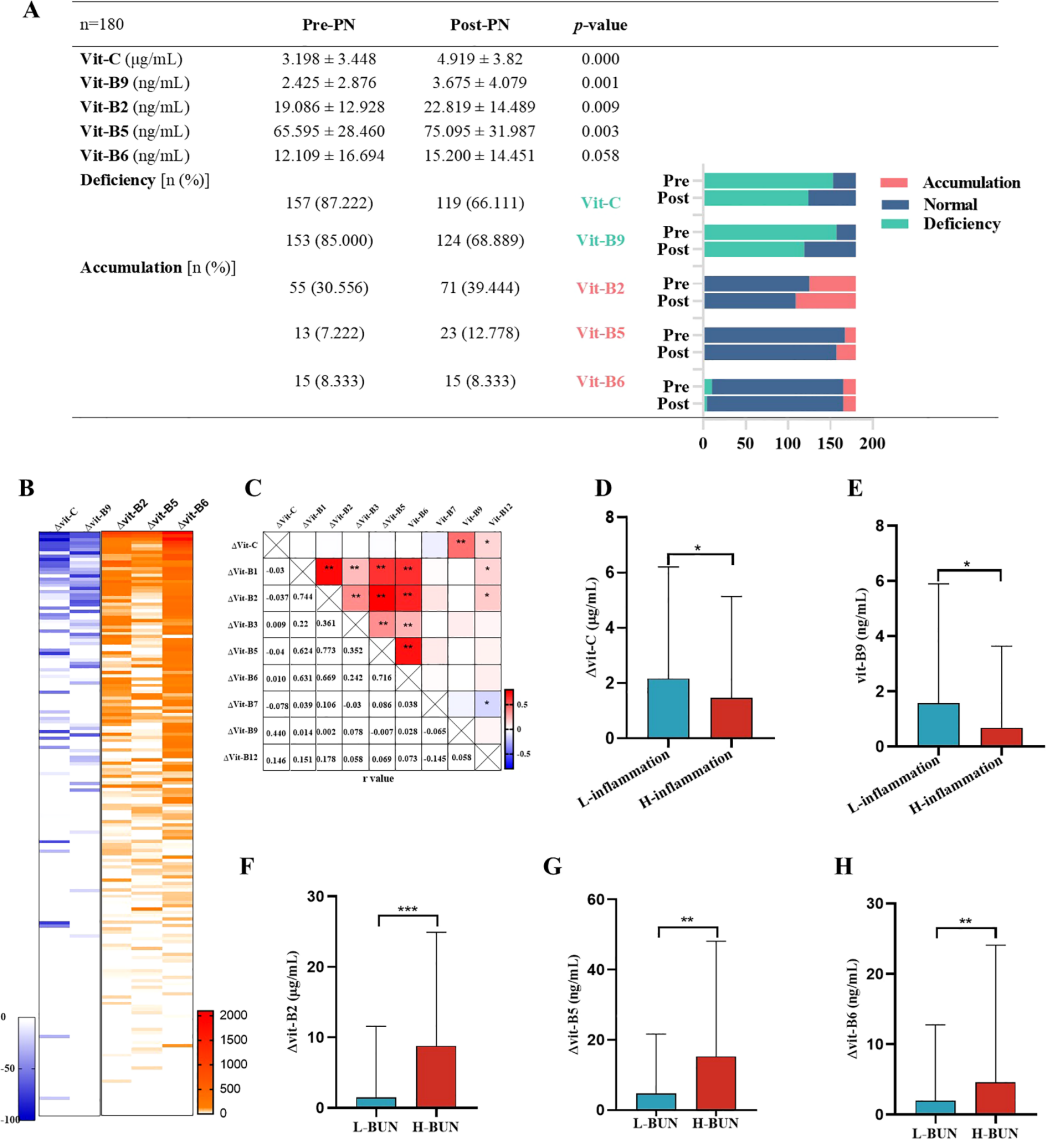
Plasma vitamin levels after PN for 1 week. n=180**(A)** Comparison of vitamin C, B9, B2, B5, and B6 levels, deficiency and accumulation rate of PN treatment. Pre-PN, PN treatment for 1 day. Post-PN, PN treatment for 7 days. **(B)** Percentage change of plasma vitamin level after PN of 180 patients. **(C)** Pairwise correlation analysis of change of plasma vitamin level after PN. **(D-F)** Inflammation inhibited the increase of vitamin C and B9 levels after PN. “L-inflammation”, no or mild grade of inflammation wit CRP<= 10 mg/L and WBC 4-10*10^9^/L; “H-inflammation”, moderate to severe grade of inflammation wit CRP>50 mg/L or WBC>15*10^9^/L. **(F-H)** Higher BUN levels accelerated the increase of vitamin B2, B5, B6 levels after PN. Reference Range of BUN: Serum, adult: 3.1-8.8mmol/L. “L-BUN” <=8.8mmol/L; “H-BUN” >8.8mmol/L.

To explore the influence of inflammation and renal dysfunction on the effects of plasma water-soluble vitamin supplementation, we monitored and compared the water-soluble vitamin levels of 180 patients on PN days 1 and 7. Vitamin C and B9 deficiency prevalence remained high over the 7 PN days even though average plasma water-soluble vitamin levels increased. The prevalence of plasma vitamin B2, B5, and B6 accumulation also increased (**Figure 5A**). Moreover, some patients demonstrated no change in plasma vitamin C and B9 levels after 7 PN days PN. In contrast, plasma vitamin B2, B5, and B6 levels increased significantly, with the rate of increase reaching 2000% (**Figure 5B**). Notably, in pairwise comparisons, changes in plasma water-soluble vitamin levels over 7 PN days were correlated. In particular, changes in plasma vitamin C and B9 levels were positively correlated; those in plasma vitamin B2, B5, and B6 levels were also positively correlated (**Figure 5C**). Furthermore, inflammation was noted to suppress increases in plasma vitamin C and B9 levels (**Figures 5D, E**). According to the GLIM criteria, 10–50 and >50 mg/L CRP may indicate moderate and severe acute inflammation, respectively (25). Renal dysfunction (i.e., high BUN levels) accelerated increases in plasma vitamin B2, B5, and B6 levels after 7 PN days (**Figures 5F–H**).

## 4 Discussion

Healthy individuals with a proper diet typically do not need additional water-soluble vitamin supplementation. In contrast, patients with an inflammatory disease or cancer have low appetite, which leads to decreases in their vitamin intake; moreover, patients with intestinal obstruction, severe pancreatitis, short bowel, or long-term gastrointestinal dysfunction- malnutrition are required to fast. The aforementioned patients require water-soluble vitamin supplementation. Nevertheless, the optimal dosage of water-soluble vitamins during illness remains unknown. The dosage of water-soluble vitamin supplementation via PN is typically determined based on the recommended dietary allowance and dietary reference intake of a healthy population (26, 27). However, water-soluble vitamin absorption, metabolism, and synthetic utilization differ between patients and healthy populations. Hence, we believe that the dosage of water-soluble vitamin supplementation should also be determined based on the disease type. To clarify plasma water-soluble vitamin levels in patients with critical digestive diseases and explore optimal vitamin supplementation dosages for these patients, we analyzed the data of plasma levels of nine water-soluble vitamins—a highlight of this study—to comprehensively reflect overall changes in water-soluble vitamins. Furthermore, we analyzed the reasons for the high incidence of vitamin disorders among hospitalized patients receiving PN. Our results provide evidence to facilitate the alleviation of water-soluble vitamin disorders and improve prognosis.

Our results showed that water-soluble vitamin abnormalities were prevalent in hospitalized patients. In particular, patients tended to be deficient in vitamin C and B9 and accumulate vitamin B2, B5, and B6. Vitamin disorders are closely related to disease occurrence (28, 29) and development (30–32). Disease results in water-soluble vitamin deficiency, which in turn accelerates disease progression and worsens prognosis. Considering the major role of water-soluble vitamins in metabolism, correcting plasma water-soluble vitamin levels is essential.

Our correlation analysis revealed that all water-soluble vitamins except vitamin B7 and B12 were associated with inflammatory markers. These results are consistent with those reported previously: inflammation does not affect plasma vitamin B7 and B12 levels. Moreover, we noted that plasma vitamin C levels decreased rapidly with the progression of inflammation, corroborating the results of Duncan et al. (33). Our univariate analysis revealed that abnormal CRP, WBC, NE, and SII were risk factors for vitamin B9 deficiency; however, only NE remained an independent risk factor after multivariate analysis. Thus, plasma vitamin B9 levels may be affected by inflammation. However, evidence suggesting that inflammation affects plasma vitamin B9 levels is lacking, mainly because studies thus far have used CRP as their inflammatory marker. A few studies have assessed the relationship of plasma vitamin B1 and B3 levels with inflammation. In general, plasma vitamin B1 and B3 levels are considered not to be affected by inflammation; however, our results demonstrated that plasma vitamin B1 levels were negatively correlated with CRP, whereas plasma vitamin B3 levels were correlated positively with WBC and NE but negatively with CRP. Vitamins B2, B3, B5, B6 was positive correlated with leukocyte/neutrophil counts, but negative correlations with CRP. Previous studies predominantly using CRP as an inflammatory marker have reported that inflammation likely reduces circulating levels of vitamins B2, B5, and B6. However, these prior investigations focused on oral intake rather than intravenous administration. In oral supplementation, inflammatory states (indicated by elevated CRP) often impair intestinal absorption, making it difficult to distinguish whether the observed vitamin depletion is driven by inflammatory mediators or malabsorption. In contrast, our study utilized peripheral infusion of water-soluble vitamins, which bypass the gastrointestinal tract and enter the bloodstream directly. Intriguingly, only vitamin B3 exhibited a significant inverse correlation with CRP. The positive associations between vitamins B2, B3, B5, B6 and leukocyte/neutrophil counts should not be simplistically interpreted as pro-inflammatory effects. While leukocytes and neutrophils directly mediate inflammation through effector functions, CRP primarily reflects systemic inflammatory intensity as a hepatic acute-phase protein. A plausible explanation lies in the metabolic roles of B vitamins: for instance, vitamin B6 serves as a coenzyme in purine synthesis and lymphocyte proliferation. Elevated leukocyte counts may reflect immune system activation, during which increased cellular metabolic demands drive vitamin redistribution to proliferating immune cells, leading to transient rises in circulating vitamin concentrations alongside immune cell expansion. Further investigations are warranted to delineate the temporal, contextual, and molecular interactions governing these associations. In general, inflammation can exacerbate vitamin C and B9 deficiency and vitamin B2, B5, and B6 accumulation. Thus, inflammatory markers should be considered when detecting plasma vitamin C, B2, B5, B6, and B9 levels and determining vitamin deficiency or accumulation status.

We screened comprehensive evaluation indicators according to the markers of inflammation, renal function, and nutritional status to predict water-soluble vitamin C and B9 deficiency and vitamin B2, B5, and B6 accumulation and then constructed corresponding models. The AUCs of these factors for predicting vitamin C deficiency, vitamin B9 deficiency, vitamin B2 accumulation, vitamin B5 accumulation, and vitamin B6 accumulation were 0.80, 0.75, 0.69, 0.79, and 0.89, respectively. In general, these factors affected not only the plasma levels of the water-soluble vitamins at a certain point but also the dynamic changes in these levels after vitamin supplementation.

Altered food intake, nutrient absorption, nutrient loss, nutrient demand, or medication use can affect plasma water-soluble vitamin levels. Moreover, plasma water-soluble vitamin levels are correlated with each other. Our heatmap revealed positive pairwise correlations between plasma water-soluble vitamin levels. In this study, we included water-soluble vitamins typically found in various water-soluble vitamin–rich foods (34). All water-soluble vitamins undergo similar metabolism (35), and they do not appear to be in a competitive relationship with each other. Notably, plasma vitamin B7 levels were observed not to be correlated with the plasma levels of other water-soluble vitamins. While inflammation shows no association with vitamin B7, its relationship with vitamin B12 remains controversial. Vitamin B7 plays a regulatory role in adaptive immunity, particularly through its modulation of T lymphocyte and NK function. However, no studies have yet demonstrated an effect of inflammation on vitamin B7 status markers, which is consistent with our results. Vitamin B12 is crucial for nerve function, DNA synthesis, and red blood cell formation. It plays a key role in homocysteine and energy metabolism. Homocysteine is linked to B12 deficiency and is associated with a higher risk of inflammatory conditions like cardiovascular and chronic kidney diseases. However, homocysteine may indirectly promote inflammation through oxidative stress and endothelial damage rather than acting directly as an inflammatory agent. Inflammation seemed to associated with elevated vitamin B12 concentrations, as demonstrated by Corcoran et al., who reported a positive correlation between CRP and cobalamin levels during the first two days of ICU admission (36, 37). Our results have not yet observed this phenomenon, and the possible reasons are as follows. European Food Safety Authority (EFSA) recommends the lowest daily requirement for vitamin B7 and B12 (among all water-soluble vitamins) to healthy adults: 40 (38) and 4 (39) μg/day, respectively. Furthermore, among all water-soluble vitamins, vitamin B7 and B12, with a narrow reference range, had the lowest plasma levels. This might be the reason that plasma vitamins B7 and B12 levels vary less among patients and are less affected by other factors. Furthermore, we propose that supplementation of water-soluble vitamins can rapidly replenish those with minimal daily needs. Renal dysfunction was noted to also affect plasma water-soluble vitamin levels. Correlation analysis revealed that plasma levels of all water-soluble vitamins, except vitamin B3 and B7, were positively correlated with renal-function markers. Thus, high BUN levels may be an independent protective factor for vitamin C and B9 deficiency, suggesting that renal dysfunction or high BUN levels might mask vitamin C and B9 deficiency. Hb was negatively correlated with plasma vitamin C levels, making altered Hb a risk factor for vitamin C deficiency. This may be because vitamin C is also present in red blood cells, and high Hb levels may represent an increase in vitamin content in red blood cells and a corresponding decrease in serum concentration. Thus, detection of vitamin content in serum and red blood cells should be performed routinely to identify vitamin deficiency. As mentioned earlier, inflammation is a risk factor for vitamin C and B9 deficiency. A high inflammation state was noted to inhibit post-PN improvements in plasma water-soluble vitamin contents. Therefore, plasma vitamin C and B9 levels should be measured with reference to renal dysfunction and inflammation, and the vitamin C and B9 supplementation dosage should be increased in inflammatory conditions.

We also assessed plasma vitamin B2, B5, and B6 accumulation—which is rarely reported, even though it can cause some ailments. Vitamin B2 accumulation may lead to the formation of potentially toxic peroxides or tryptophan-riboflavin adducts that have hepatorenal toxicity (40). Moreover, vitamin B5 accumulation may lead to diarrhea and muscle pain, whereas vitamin B6 accumulation may cause sensory neuropathy (41, 42), cutaneous impairment, and dermatologic lesions (43). Vitamin B2, B5, and B6 accumulation together may increase when inflammation and renal dysfunction. However, if water-soluble vitamin supplementation is continued at this time, the consequences can be imagined. Therefore, in patients with high inflammation and renal dysfunction, water-soluble vitamins should be supplemented with caution.

This study has some limitations, which may be resolved as follows: First, future prospective studies are warranted to validate these observations and clarify causal relationships. Second, because vitamins are affected by many factors, combined analysis of emerging parameters—fluid balance, medication utilization, and related factors—is essential to predict vitamin deficiency and accumulation. Third, relevant randomized controlled trials on diseases in parts other than the gastrointestinal tract may be conducted. Finally, considering the importance of our findings, research on newer molecular mechanisms underlying the changes we noted in the plasma water-soluble vitamin levels is warranted.

## 5 Conclusions

Vitamin disorders, including deficiency and accumulation, are caused by numerous factors, particularly insufficient dietary vitamin intake, inflammation, and renal dysfunction. Hospitalized patients typically experience varying degrees of abnormalities in dietary vitamin intake, inflammation, and nephrotoxic drug use; this increases vitamin disorder incidence. To promote recovery and correct plasma vitamin levels among hospitalized patients, treatment should target not only the primary disorder or condition but also water-soluble vitamin abnormalities. For patients with high inflammation, vitamin C and B9 supplementation should surpass standard recommended intake levels. In cases of renal impairment, avoid indiscriminate supplementation of vitamins B2, B5, and B6. If supplementation is necessary, consider alternate-day dosing or reduce daily dosages below guideline levels to ensure safety.

## Data Availability

The original contributions presented in the study are included in the article/**Supplementary Material**. Further inquiries can be directed to the corresponding authors.
