# Supplementary material for: Individualized supplement of water-soluble vitamins: the influence of inflammation and renal function on circulating concentrations in critically digestive disease patients
Source: Front Immunol. 2025 Jul 25;16:1583568. doi: 10.3389/fimmu.2025.1583568 (PMC12331502; doi:10.3389/fimmu.2025.1583568)
Supplement: Supplementary file 1 [file DataSheet1.docx]

**Table S1** Univariate logistic regression analysis of Vit-C deficiency

| **Vit-C** | **Normal group**  n=97 | **Deficiency group**  n=381 | **Univariate Analysis** | |
| --- | --- | --- | --- | --- |
|  |  |  | OR (95%CI) | P-Value |
| Age, years, median [IQR] | 66.500(61.500-75.750) | 66.000(59.000-73.250) | 0.976(0.956-0.997) | **0.026** |
| Men, n (%) | 61(62.900) | 262(68.800) | 1.299(0.816-2.070) | 0.270 |
| **Renal function index** |  |  |  |  |
| BUN（mmol/L） | 9.305(6.723-12.960) | 6.990(5.245-11.095) | 0.952(0.919-0.986) | **0.006** |
| Cr（μmol/L） | 69.000(44.750-93.750) | 61.500(48.000-89.750) | 0.998(0.996-1.001) | 0.247 |
| UA（μmol/L） | 169.500(78.250-279.250) | 128.500(93.750-173.250) | 0.998(0.996-1.000) | 0.072 |
| Urine volume/d（mL） | 1975.000(1531.250-2625.000) | 1865.000(1240.000-2662.500) | 1.000(1.000-1.000) | 0.126 |
| **Inflammatory biomarkers** |  |  |  |  |
| WBC（×10^9^/L） | 7.125(4.330-9.068) | 9.745(6.913-12.948) | 1.107(1.043-1.175) | **0.001** |
| NE（×10^9^/L） | 5.680(3.403-7.515) | 8.220(5.418-11.713) | 1.162(1.082-1.247) | **0.000** |
| SII | 1176.245(529.268-2336.175) | 1730.109(975.949-3250.989) | 1.000(1.000-1.001) | **0.000** |
| CRP（μg/mL） | 58.050(24.450-82.225) | 102.055(0.950-162.250) | 1.015(1.007-1.022) | **0.000** |
| **Nutritional status** |  |  |  |  |
| LYM（×10^9^/L） | 0.805(0.433-1.113) | 0.740(0.508-1.080) | 0.900(0.781-1.038) | 0.148 |
| Hb（g/L） | 85.500(76.250-97.750) | 95.500(83.000-110.000) | 1.025(1.012-1.038) | **0.000** |
| ALB（mg/dL） | 32.050(29.025-35.475) | 32.200(28.975-36.025) | 0.972(0.939-1.006) | 0.099 |
| PA（mg/dL） | 10.050(6.300-15.573) | 9.000(5.715-12.625) | 1.016(0.974-1.061) | 0.457 |
| **Diseases type** [n(%)] |  |  |  |  |
| Esophageal disease | 4(4.100) | 12(3.100) | 1.000 |  |
| Gastric disease | 16(16.500) | 40(10.500) | 0.736(0.204-2.657) | 0.640 |
| Intestines problem | 36(37.100) | 113(29.700) | 0.613(0.265-1.419) | 0.253 |
| Hepatobiliary disease | 28(28.900) | 163(42.800) | 0.770(0.377-1.571) | 0.472 |
| Pancreatic Disease | 13(13.400) | 53(13.900) | 1.428(0.690-2.955) | 0.337 |

**Table S2** Univariate logistic regression analysis of Vit-B9 deficiency

| **Vit-B9** | **Normal group**  n=102 | **Deficiency group**  n=375 | **Univariate Analysis** | |
| --- | --- | --- | --- | --- |
|  |  |  | OR (95%CI) | *P-*Value |
| Age, years, median [IQR] | 67.00(60.500-80.500) | 66.000(58.250-72.000) | 0.972(0.952-0.993) | **0.008** |
| Men, n (%) | 58(56.900) | 265(70.700) | 1.828(1.165-2.868) | **0.009** |
| **Renal function index** |  |  |  |  |
| BUN（mmol/L） | 9.700(6.160-15.460) | 6.865(5.343-10.755) | 0.946(0.913-0.980) | **0.002** |
| Cr（μmol/L） | 69.000(47.000-101.250) | 62.000(48.000-85.500) | 0.999(0.997-1.002) | 0.566 |
| UA（μmol/L） | 167.000(102.250-318.000) | 128.000(86.750-172.750) | 0.998(0.996-1.000) | **0.042** |
| Urine volume/d（mL） | 1892.500(1377.500-2400.000) | 1890.000(1252.250-2687.500) | 1.000(1.000-1.000) | 0.326 |
| **Inflammatory biomarkers** |  |  |  |  |
| WBC（×10^9^/L） | 6.795(5.158-10.253) | 9.735(7.008-13.163) | 1.088(1.029-1.150) | **0.003** |
| NE（×10^9^/L） | 5.670(3.673-8.295) | 8.275(5.523-11.718) | 1.212(0.820-1.792) | 0.335 |
| SII | 1189.353(4569.631-2526.474) | 1842.308(986.768-3235.235) | 1.000(1.000-1.000) | **0.028** |
| CRP（μg/mL） | 68.500(39.475-104.500) | 99.000(43.125-158.750) | 1.006(1.001-1.010) | **0.019** |
| **Nutritional status** |  |  |  |  |
| LYM（×10^9^/L） | 0.800(0.555-1.195) | 0.860(0.570-1.190) | 1.110(1.041-1.182) | **0.001** |
| Hb（g/L） | 90.500(79.750-100.000) | 95.000(82.000-109.000) | 1.024(1.012-1.037) | **0.000** |
| ALB（mg/dL） | 31.850(29.550-34.525) | 32.450(28.825-35.950) | 1.016(0.974-1.061) | 0.450 |
| PA（mg/dL） | 9.050(5.175-13.900) | 9.050(5.900-12.675) | 1.006(0.970-1.042) | 0.764 |
| **Diseases type** [n(%)] |  |  |  |  |
| Esophageal disease | 2(1.961) | 14(3.733) | 1.000 |  |
| Gastric disease | 13(12.745) | 43(11.467) | 1.556(0.311-7.768) | 0.590 |
| Intestines problem | 45(44.118) | 103(27.467) | 0.735(0.305-1.774) | 0.493 |
| Hepatobiliary disease | 30(29.412) | 161(42.933) | 0.509(0.248-1.042) | 0.065 |
| Pancreatic Disease | 12(11.765) | 54(14.400) | 1.193(0.571-2.492) | 0.640 |

**Table S3** Univariate logistic regression analysis of Vit-B2 accumulation.

| **Vit-B2** | **Normal group**  n=309 | **Accumulation group**  n=165 | **Univariate Analysis** | |
| --- | --- | --- | --- | --- |
|  |  |  | OR (95%CI) | *P-*Value |
| Age, years, median [IQR] | 65.000(56.500-72.000) | 67.000(63.000-76.000) | 1.015(0.997-1.032) | 0.096 |
| Men, n (%) | 210(68.000) | 110(66.700) | 0.943(0.631-1.410) | 0.774 |
| **Renal function index** |  |  |  |  |
| BUN（mmol/L） | 6.320(5.160-9.355) | 10.440(7.380-15.480) | 1.145(1.095-1.196) | **0.000** |
| Cr（μmol/L） | 58.000(47.500-76.000) | 83.000(47.000-130.000) | 1.007(1.003-1.011) | **0.000** |
| UA（μmol/L） | 125.000(87.500-165.500) | 163.000(120.000-318.000) | 1.004(1.002-1.006) | **0.000** |
| Urine volume/d（mL） | 1900.000(1300.000-2735.000) | 1850.000(1200.000-2500.000) | 0.9998(0.9995-0.9999) | **0.040** |
| **Inflammatory biomarkers** |  |  |  |  |
| WBC（×10^9^/L） | 9.270(6.125-12.515) | 8.720(6.750-12.290) | 1.029(0.993-1.065) | 0.113 |
| NE（×10^9^/L） | 7.590(4.850-11.275) | 6.970(5.370-10.250) | 1.007(0.979-1.036) | 0.631 |
| SII | 95.800(39.350-150.500) | 78.100(41.900-137.000) | 1.000(1.000-1.000) | 0.563 |
| CRP（μg/mL） | 9.270(6.125-12.515) | 8.720(6.750-12.290) | 0.998(0.994-1.001) | 0.206 |
| **Nutritional status** |  |  |  |  |
| LYM（×10^9^/L） | 1862.229(979.760-3221.369) | 1210.000(826.634-2936.1406) | 1.126(0.946-1.340) | 0.182 |
| Hb（g/L） | 96.000(83.500-109.000) | 90.000(78.000-103.000) | 0.990(0.980-0.999) | **0.036** |
| ALB（mg/dL） | 32.600(29.450-36.150) | 31.800(28.200-34.600) | 0.944(0.909-0.980) | **0.003** |
| PA（mg/dL） | 9.200(5.850-12.550) | 9.000(5.700-14.600) | 0.997(0.967-1.028) | 0.863 |
| **Diseases type** [n(%)] |  |  |  |  |
| Esophageal disease | 14(4.531) | 2(1.212) | 1.000 |  |
| Gastric disease | 39(12.621) | 17(10.303) | 0.429(0.088-2.093) | 0.295 |
| Intestines problem | 86(27.832) | 63(38.182) | 1.308(0.586-2.919) | 0.513 |
| Hepatobiliary disease | 122(39.482) | 67(40.606) | 2.198(1.144-4.220) | **0.018** |
| Pancreatic Disease | 48(15.534) | 16(9.697) | 1.648(0.869-3.123) | 0.126 |

**Table S4** Univariate logistic regression analysis of Vit-B5 accumulation

| **Vit-B5** | **Normal group**  n=419 | **Accumulation group**  n=58 | **Univariate Analysis** | |
| --- | --- | --- | --- | --- |
|  |  |  | OR (95%CI) | *P-*Value |
| Age, years, median [IQR] | 66.000(58.500-72.000) | 70.000(64.000-79.000) | 1.032(1.005-1.059) | **0.021** |
| Men, n (%) | 287(68.500) | 36(62.100) | 0.753(0.426-1.330) | 0.328 |
| **Renal function index** |  |  |  |  |
| BUN（mmol/L） | 6.840(5.250-10.095) | 13.550(9.810-19.080) | 1.152(1.102-1.204) | **0.000** |
| Cr（μmol/L） | 60.000(47.000-79.000) | 98.000(72.000-180.000) | 1.008(1.004-1.011) | **0.000** |
| UA（μmol/L） | 128.000(84.000-172.500) | 274.000(135.500-445.000) | 1.007(1.005-1.010) | **0.000** |
| Urine volume/d（mL） | 1950.000(1317.500-2735.000) | 1500.000(827.500-2000.000) | 0.999(0.999-0.9997) | **0.000** |
| **Inflammatory biomarkers** |  |  |  |  |
| WBC（×10^9^/L） | 8.610(6.465-12.290) | 10.560(6.970-15.600) | 1.071(1.025-1.118) | **0.002** |
| NE（×10^9^/L） | 6.930(5.055-10.185) | 9.190(5.985-14.210) | 1.030(0.996-1.066) | 0.082 |
| SII | 1620.000(911.249-2792.902) | 1499.842(782.342-4190.642) | 1.000(1.000-1.000) | 0.891 |
| CRP（μg/mL） | 91.200(41.250-145.500) | 69.200(31.000-134.000) | 0.997(0.992-1.002) | 0.253 |
| **Nutritional status** |  |  |  |  |
| LYM（×10^9^/L） | 0.770(0.495-1.135) | 0.670(0.480-0.995) | 1.089(0.954-1.242) | 0.207 |
| Hb（g/L） | 95.000(82.500-107.500) | 91.000(77.500-101.500) | 0.991(0.978-1.006) | 0.232 |
| ALB（mg/dL） | 32.300(29.400-35.750) | 31.600(27.700-35.200) | 0.914(0.863-0.968) | **0.002** |
| PA（mg/dL） | 9.200(5.900-12.750) | 7.600(4.650-18.550) | 0.969(0.922-1.018) | 0.206 |
| **Diseases type [n(%)]** |  |  |  |  |
| Esophageal disease | 15(3.600) | 1(1.700) | 1.000 |  |
| Gastric disease | 52(12.400) | 4(6.900) | 0.813(0.088-7.489) | 0.855 |
| Intestines problem | 117(27.900) | 31(53.400) | 0.938(0.239-3.678) | 0.927 |
| Hepatobiliary disease | 174(41.500) | 17(29.300) | 3.232(1.196-8.734) | **0.021** |
| Pancreatic Disease | 61(14.600) | 5(8.600) | 1.192(0.422-3.369) | 0.740 |

**Table S5** Univariate logistic regression analysis of Vit-B6 accumulation

| **Vit-B6** | **Normal group**  n=425 | **Accumulation group**  n=53 | **Univariate Analysis** | |
| --- | --- | --- | --- | --- |
|  |  |  | OR (95%CI) | *P-*Value |
| Age, years, median [IQR] | 65.500(58.250-72.000) | 75.000(64.500-78.750) | 1.039(1.010-1.069) | **0.008** |
| Men, n (%) | 288(67.800) | 35(66.000) | 0.925(0.506-1.692) | 0.800 |
| **Renal function index** |  |  |  |  |
| BUN（mmol/L） | 6.77(5.235-9.450) | 17.28511.895-22.1 | 1.231(1.166-1.299) | **0.000** |
| Cr（μmol/L） | 58.000(45.250-77.000) | 177.500(96.250-309.250) | 1.018(1.013-1.024) | **0.000** |
| UA（μmol/L） | 125.000(84.250-166.750) | 345.000(179.500-469.250) | 1.011(1.008-1.014) | **0.000** |
| Urine volume/d（mL） | 2000.000(1400.000-2700.000) | 890.000(277.500-1887.500) | 0.999(0.998-0.999) | **0.000** |
| **Inflammatory biomarkers** |  |  |  |  |
| WBC（×10^9^/L） | 8.545(6.315-12.175) | 10.815(7.563-15.113) | 1.051(1.006-1.099) | **0.026** |
| NE（×10^9^/L） | 6.895(4.960-10.035) | 9.405(5.843-13.368) | 1.023(0.989-1.058) | 0.185 |
| SII | 1628.151(907.453-2803.253) | 1367.418(755.093-3769.688) | 1.000(1.000-1.000) | 0.921 |
| CRP（μg/mL） | 83.330(9.125-137.750) | 88.840(5.550-182.750) | 1.002(0.998-1.006) | 0.352 |
| **Nutritional status** |  |  |  |  |
| LYM（×10^9^/L） | 0.780(0.525-1.143) | 0.680(0.393-0.883) | 1.082(0.947-1.236) | 0.249 |
| Hb（g/L） | 95.000(83.000-107.750) | 82.000(77.000-100.000) | 0.987(0.972-1.002) | 0.084 |
| ALB（mg/dL） | 32.350(29.325-36.075) | 31.850(28.650-34.250) | 0.935(0.883-0.991) | **0.023** |
| PA（mg/dL） | 9.200(5.910-12.875) | 7.400(4.800-13.225) | 0.976(0.929-1.027) | 0.351 |
| **Diseases type [n(%)]** |  |  |  |  |
| Esophageal disease | 16(3.765) | 1(1.887) | 1.000 |  |
| Gastric disease | 51(12.000) | 5(9.434) | 0.453(0.530-3.895) | 0.471 |
| Intestines problem | 126(29.647) | 22(41.509) | 0.711(0.219-2.311) | 0.570 |
| Hepatobiliary disease | 174(40.941) | 17(32.075) | 1.266(0.532-3.072) | 0.594 |
| Pancreatic Disease | 58(13.647) | 8(15.094) | 0.708(0.290-1.727) | 0.448 |

Key findings are highlighted in bold for significant results in Tables S1-S5.
